# Cybercontrol and Cyberaggression in Young Adults’ Romantic Relationships and Their Associations with Psychological Distress and Romantic Love Myths

**DOI:** 10.3390/healthcare14183056

**Published:** 2026-09-17

**Authors:** Alejandro Arévalo-Martínez, María Alonso-Santos, Cristina Pinar-Gómez, Carlos Barbosa-Torres, María Pilar Cantillo-Cordero, Juan Manuel Moreno-Manso

**Affiliations:** Department of Psychology, Faculty of Education and Psychology, University of Extremadura, 06006 Badajoz, Spain; malonsohs@alumnos.unex.es (M.A.-S.); cpinargo@alumnos.unex.es (C.P.-G.); carlosbarbosa@unex.es (C.B.-T.); pcantillo@unex.es (M.P.C.-C.); jmmanso@unex.es (J.M.M.-M.)

**Keywords:** digital dating violence, cybercontrol, psychological abuse, romantic love myths, psychological distress, young adults

## Abstract

**Highlights:**

**What are the main findings?**

- Women reported greater psychological distress and higher scores on several dimensions of digital dating violence, whereas men showed stronger endorsement of romantic love myths.
- Formal comparisons indicated that the interaction between psychological distress and romantic love myths differed by sex for victimization–cyberaggression, perpetration–cybercontrol, and total victimization, independently of problematic social media use.

**What are the implications of the main findings?**

- Romantic love myth endorsement appears to be relevant to the pattern of association between psychological distress and cybercontrol and cyberaggression within intimate relationships.
- Prevention programs should challenge beliefs that normalize digital surveillance and control and improve recognition of cybercontrol as a form of violence, while considering its documented associations with other forms of intimate partner violence.

**Abstract:**

**Background/Objectives**: Digital technologies have transformed how young adults in romantic relationships communicate, develop intimacy, and manage control. Although these technologies facilitate connection, they may enable surveillance, control, and aggression. This study examined associations of psychological distress and romantic love myths with digital dating violence while controlling for problematic social media use. **Methods**: This observational, cross-sectional study included 336 university students aged 18–25 years. Participants completed the Cyber-Violence in Adolescent Couples Scale, adapted for young adults (Cib-VPA), the Depression Anxiety Stress Scales-21 (DASS-21), the Scale of Myths of Romantic Love (SMRL), and the Bergen Social Media Addiction Scale (BSMAS). Differences between men and women, partial correlations, pooled regression models testing the Sex × DASS-21 × SMRL interaction, and sex-stratified regression models were examined. **Results**: Women reported higher levels of anxiety, depression, stress, psychological distress, cybercontrol victimization, cyberaggression victimization, cybercontrol perpetration, and total victimization. Men showed stronger endorsement of romantic love myths. Formal pooled comparisons indicated that the DASS-21 × SMRL interaction differed by sex for victimization–cyberaggression, perpetration–cybercontrol, and total victimization. Sex-stratified models showed significant interactions for several dimensions of digital dating violence. Simple slopes showed that psychological distress was positively associated with total perpetration among men and women and with total victimization among women when romantic love myth endorsement was high. **Conclusions**: Psychological distress and romantic love myths appear to be associated with digital dating violence. Prevention programs should support psychological well-being, challenge romantic love myths, and improve recognition of cybercontrol as a form of intimate partner violence.

## 1. Introduction

Romantic relationships among young people now develop within a context of high digital connectivity, in which dynamics of intimacy, communication, and control may extend into online environments. Although digital technologies facilitate continuous connection and help maintain romantic bonds, they may also increase opportunities for monitoring, harassment, and control within intimate relationships [1,2,3]. These behaviors are particularly relevant because they occur within relationships characterized by intimacy, trust, and emotional dependence and may become embedded in less visible forms of psychological abuse and coercive control, including surveillance, intimidation, and the progressive restriction of a partner’s autonomy [4,5,6].

In this context, digital violence in intimate relationships, also referred to as cyber dating abuse, encompasses abusive behaviors carried out through digital media with the aim of controlling, harming, intimidating, or monitoring a current or former partner [7,8]. These behaviors are generally grouped into two main dimensions: cyberaggression, which includes direct acts of harm such as insults, threats, humiliation, or the dissemination of private information; and cybercontrol, which refers to monitoring behaviors such as checking a partner’s mobile phone, demanding passwords, supervising interactions on social media, or requiring constant availability [9,10]. These behaviors may involve both victimization and perpetration, roles that may overlap in young relationships and make it difficult to draw a rigid distinction between those who perpetrate and those who experience violence [7,11]. This distinction is relevant because digital abuse rarely occurs in isolation; rather, it may coexist with offline forms of intimate partner violence and has also been associated with physical, psychological, or coercive violence in intimate relationships and domestic settings [8,11,12,13].

Among the factors that may contribute to these dynamics, psychological distress appears to play an important role [14,15]. Previous research has linked digital dating violence to higher levels of depression, anxiety, and general psychological symptoms, particularly among individuals who experience online or offline victimization [14,15,16,17]. In addition, factors such as attachment insecurity and emotional dependence may increase involvement in digital control dynamics, particularly when surveillance is used as a maladaptive strategy to cope with fear of abandonment [18]. Within this framework, problematic social media use has also been associated with greater psychological distress and experiences of dating violence [19,20].

Alongside these factors, romantic love myths may contribute to the normalization of abusive behaviors within intimate relationships [21,22]. This belief system idealizes love and promotes a view of relationships based on self-sacrifice, exclusivity, and emotional fusion with one’s partner, thereby attributing a positive meaning to jealousy, possessiveness, or control within the romantic bond [23]. In digital environments, these myths may lead intrusive practices, such as checking a partner’s phone, monitoring social media activity, or demanding constant availability, to be interpreted as expressions of care or romantic interest rather than recognized as forms of abuse [21,22]. From this perspective, emotional dependence or fear of abandonment may be associated with greater involvement in digital monitoring and control [18], while jealousy has also been linked to online dating violence [24]. Romantic love myths may provide a cognitive framework in which such controlling or intrusive behaviors are more readily normalized or justified [21,22,25]. Consequently, the association between psychological distress and digital dating violence may be stronger when endorsement of these beliefs is high.

Previous research has identified sex-related differences in both the experience and emotional consequences of digital dating violence [16], as well as in the endorsement of romantic love myths [22]. In addition, sex-specific patterns have been reported in the psychosocial and relational factors associated with cybercontrol and cyberaggression, including romantic myths, sexist attitudes, and offline dating violence [26,27].

Despite growing interest in digital violence among young adults in romantic relationships, evidence regarding the combined role of psychological distress and romantic love myths in these dynamics remains limited, particularly when distinguishing between victimization and perpetration and between cybercontrol and cyberaggression [7,14,22,28]. Therefore, the aims of the present study were to: (1) describe levels of digital dating violence, psychological distress, romantic love myths, and problematic social media use among young people; (2) examine differences between men and women in the study variables; (3) analyze the associations of psychological distress and romantic love myths with digital dating violence; and (4) examine whether the interaction between psychological distress and romantic love myths is associated with digital dating violence and whether this interaction differs between men and women, while adjusting for problematic social media use.

## 2. Materials and Methods

The reporting of this observational, cross-sectional study followed the recommendations of the STROBE Statement for cross-sectional studies [29].

### 2.1. Participants

The study included 336 university students aged 18–25 years who were enrolled in different degree programs at the University of Extremadura, Spain. Data were collected in 2026 at the Faculty of Education and Psychology, the Faculty of Medicine and Health Sciences, and the Faculty of Sciences of the University of Extremadura using a non-probability sampling method. The selected age range was based on the conceptualization of emerging adulthood as a developmental stage occurring approximately between 18 and 25 years of age, characterized by the transition toward autonomy and the progressive exploration and consolidation of romantic relationships [30,31]. This developmental period is particularly relevant to the study of digital dating violence because digital technologies play a central role in communication, intimacy, and the management of romantic relationships among young people and may facilitate dynamics of control, surveillance, and cyberaggression [32,33].

The inclusion criteria were enrollment at the University of Extremadura, being within the specified age range, currently being in a romantic relationship or having been in one during the previous 12 months, and voluntarily agreeing to participate by providing written informed consent. Participants were excluded if questionnaires were completed incorrectly, response patterns were inconsistent, relevant sociodemographic or study-related information was missing, or the inclusion criteria were not met.

### 2.2. Instruments

1. Bergen Social Media Addiction Scale (BSMAS) [19]. This instrument assesses problematic social media use during the previous year. It comprises six items rated on a 5-point Likert scale (1 = very rarely, 2 = rarely, 3 = sometimes, 4 = often, and 5 = very often). Higher scores indicate a greater frequency of behaviors associated with problematic or addictive social media use. Regarding its psychometric properties, the Spanish version has demonstrated good internal consistency, with α = 0.85 [34]. In the present study, internal consistency was adequate, with α = 0.79.
2. Depression Anxiety Stress Scales-21 (DASS-21) [35]. This instrument assesses symptoms of depression, anxiety, and stress. It comprises 21 items rated on a 4-point Likert scale (0 = did not apply to me at all, 1 = applied to me to some degree or some of the time, 2 = applied to me to a considerable degree or a good part of the time, and 3 = applied to me very much or most of the time). Each subscale score was multiplied by two to apply the conventional cutoff scores, and the total score was calculated by summing the three transformed subscale scores [35]. Symptom severity is classified into five categories: normal, mild, moderate, severe, and extremely severe [35,36]. The total score was analyzed as a continuous variable in the inferential analyses, whereas the conventional severity categories were used only for descriptive interpretation of the subscale scores. The Spanish version has demonstrated good internal consistency, with values ranging from α = 0.73 to α = 0.81 across its subscales [37]. In the present study, internal consistency was adequate for all scores, with α = 0.78 for stress, α = 0.80 for anxiety, α = 0.79 for depression, and α = 0.92 for the total score.
3. Scale of Myths of Romantic Love (SMRL) [38]. This instrument assesses the degree of endorsement of stereotypical beliefs about romantic relationships. It comprises 11 items rated on a 5-point Likert scale (1 = strongly disagree, 2 = disagree, 3 = neither agree nor disagree, 4 = agree, and 5 = strongly agree). The scale assesses the endorsement of traditional myths about love that may contribute to the normalization of abusive relationship dynamics. It comprises two dimensions: idealized love and distorted love. Higher scores indicate stronger endorsement of romantic love myths. The Spanish version has demonstrated adequate internal consistency, with α = 0.70 [38]. In the present study, internal consistency for the total score was adequate, with α = 0.72.
4. Cyber-Violence in Adolescent Couples Scale, adapted for young adults (Cib-VPA) [39,40]. This instrument assesses digital violence within romantic relationships from both perpetration and victimization perspectives. Although it was initially developed for adolescents, Redondo et al. [40] examined its psychometric properties in Spanish young adults and proposed a version with adequate evidence of validity and reliability. This adapted version was used in the present study. It comprises 18 items rated on a 4-point Likert scale (1 = never, 2 = sometimes, 3 = usually, and 4 = always) and assesses behaviors perpetrated toward or experienced from a current or former partner in digital environments during the previous year. The instrument includes two subscales: perpetrated cyber abuse, which assesses aggressive and controlling behaviors directed toward a partner through digital media, and cybervictimization, which assesses the experience of these same behaviors. Both subscales comprise two dimensions: cyberaggression and cybercontrol. Higher scores indicate a greater frequency of the assessed behaviors. The version adapted for Spanish young adults has demonstrated adequate evidence of validity and internal consistency, with McDonald’s omega coefficients of ω = 0.77 for total cybervictimization and ω = 0.72 for total perpetrated cyber abuse [40]. In the present study, internal consistency was adequate for the overall scores, with α = 0.88 for total cybervictimization, α = 0.73 for total perpetrated cyber abuse, and α = 0.83 for the full scale.

### 2.3. Procedure

Students at the University of Extremadura were recruited through direct invitations in classroom settings and completed a battery of questionnaires. Before the assessment began, participants were informed about the aims of the study, the voluntary nature of their participation, the confidentiality of the data, and their right to withdraw from the study at any time without consequences. All participants provided written informed consent before completing the questionnaires. The questionnaires were administered in paper-and-pencil format collectively in the classroom but completed individually, with an approximate completion time of 30 min. To ensure consistency in administration, all assessors received prior training in the administration of the instruments and remained present throughout the assessment to answer questions without influencing participants’ responses. No incidents were recorded during data collection.

All procedures complied with the ethical guidelines of the University of Extremadura (Ref.: 92/2020) and with the principles of the Declaration of Helsinki and its subsequent amendments. The study also complied with the requirements of Spanish Organic Law 3/2018 of 5 December on the Protection of Personal Data and Guarantee of Digital Rights, ensuring the anonymity, confidentiality, and secure storage of the information provided by participants.

### 2.4. Data Analysis

Data processing and statistical analyses were conducted using IBM SPSS Statistics for Windows, version 27.0 (IBM Corp., Armonk, NY, USA). First, descriptive analyses were performed by calculating frequencies and percentages for categorical variables and means and standard deviations for continuous variables. Independent-samples *t* tests were used to compare men and women on problematic social media use, psychological distress, romantic love myths, and the dimensions of digital dating violence. Welch’s correction was applied when the assumption of homogeneity of variances was not met. The magnitude of between-group differences was estimated using Hedges’ *g* and its 95% confidence interval. Positive values indicated higher scores among women, whereas negative values indicated higher scores among men. No missing data were identified in the variables included in the analyses; therefore, all participants were included in the corresponding analyses.

Subsequently, the associations of psychological distress and romantic love myths with digital dating violence were examined using partial Pearson correlations while controlling for problematic social media use. These analyses were conducted separately for men and women. To formally evaluate whether the DASS-21 × SMRL interaction differed between men and women, pooled linear regression models were estimated for each digital dating violence outcome using the total sample. These models included BSMAS, sex, DASS-21, SMRL, all constituent two-way interactions involving sex, DASS-21, and SMRL, the Sex × DASS-21 × SMRL interaction, and the Sex × BSMAS interaction. Sex was coded as 0 = men and 1 = women; therefore, the coefficient for the three-way interaction represented the difference in the DASS-21 × SMRL interaction between women and men. Given the theoretical rationale described in the Introduction, sex-stratified linear regression analyses were subsequently estimated to characterize the associations within each group while controlling for problematic social media use.

The sex-stratified models simultaneously included problematic social media use as a covariate, DASS-21 and SMRL scores as main effects, and the DASS-21 × SMRL interaction term. DASS-21 and SMRL scores were mean-centered within each sex before calculating the interaction terms. Because the sex-stratified regression models were estimated separately by sex, within-sex centering was used so that zero corresponded to the sex-specific mean, facilitating the interpretation of the DASS-21 and SMRL coefficients within each stratified model. The same scores centered within sex were used in the pooled models to ensure comparability with the sex-stratified analyses. Within the sex-stratified models, Δ*R*^2^ was calculated for each outcome as the increase in *R*^2^ from a reduced model containing BSMAS and the two mean-centered main effects to the corresponding full model that additionally included the DASS-21 × SMRL interaction term. Δ*R*^2^ was interpreted as an index of the incremental magnitude of the interaction effect and was reported with its corresponding 95% confidence interval, estimated using a modified percentile bootstrap with 10,000 resamples based on the procedure described by Algina et al. [41] and adapted to the HC3 inferential framework used in the present analyses. The lower confidence limit was set to zero when the corresponding HC3 interaction test was nonsignificant at α = 0.05. Regression assumptions were examined through residual analyses, including normality, homoscedasticity, linearity, and independence. Potentially influential cases were assessed using Cook’s distance, and multicollinearity was evaluated using variance inflation factors. HC3 heteroscedasticity-consistent standard errors were used for statistical inference in both the pooled and sex-stratified regression models and in the simple slope analyses. Interaction plots and simple slope analyses were conducted for the sex-stratified total-score models in which the DASS-21 × SMRL interaction remained significant after correction for multiple comparisons.

To control for multiple comparisons and reduce the risk of Type I errors, the Benjamini–Hochberg procedure [42] was applied using a false discovery rate of 0.05 to predefined sets of related tests. The 14 comparisons between men and women reported in Table 1 were adjusted together. For the partial correlation analyses, adjustment was performed separately across the six digital dating violence outcomes for each predictor (DASS-21 and SMRL) within each sex. For the pooled regression analyses, adjustment was performed across the six Sex × DASS-21 × SMRL interaction tests. For the sex-stratified regression analyses, adjustment was performed separately across the six DASS-21 × SMRL interaction tests within each sex. Other *p*-values, including the between-sex comparison of age, regression coefficients other than the focal Sex × DASS-21 × SMRL interaction terms in the pooled models and the DASS-21 × SMRL interaction terms in the sex-stratified models, and overall model tests, were reported without adjustment because they were not primary targets of the multiple-comparison analyses: age was included for sample characterization, the remaining regression coefficients for model interpretation, and the overall tests to assess model-level significance. Simple slope analyses were conducted only as follow-up probes of total-score interactions that remained significant after Benjamini–Hochberg correction and were therefore reported without additional adjustment. Statistical significance was set at *p* < 0.05.

To justify the sample size, an a priori power analysis was conducted for linear regression models with four predictors, assuming a small effect size according to Cohen’s criteria [43], *f*^2^ = 0.04, a significance level of α = 0.05, and a desired statistical power of 1 − β = 0.80. The analysis indicated that an approximate sample size of 300 participants was required; therefore, the final sample of 336 participants was considered adequate for the main study objectives. In addition, the total sample size was comparable to those used in previous studies on digital dating violence among young people [27,44]. Because the sex-stratified interaction models were estimated separately for men and women, sensitivity analyses were also conducted for the incremental effect of the interaction term, considering one tested predictor within models containing four predictors in total. These analyses indicated that the available sample sizes allowed the detection of minimum effect sizes of *f*^2^ = 0.064 among men and *f*^2^ = 0.037 among women.

## 3. Results

The study included 336 participants aged 18–25 years, all of whom were students enrolled at the University of Extremadura. The sample comprised 124 men (36.90%) and 212 women (63.10%). The mean age was 22.38 years (SD = 1.75) among men and 22.14 years (SD = 1.71) among women, with no statistically significant difference between the groups (*p* = 0.215). The mean duration of the current or most recent romantic relationship was 23.4 months (SD = 18.6) in the total sample, 22.1 months (SD = 17.8) among men, and 24.2 months (SD = 19.1) among women. No missing data were identified in the variables included in the analyses.

The results of the independent-samples *t* tests for the BSMAS, DASS-21, SMRL, and Cib-VPA are presented in Table 1. No statistically significant differences between men and women were found for BSMAS scores, although women had a slightly higher mean score. In both groups, mean scores were below the theoretical midpoint of the scale, suggesting low levels of problematic social media use.

Regarding the DASS-21, statistically significant differences were observed in all dimensions, with women reporting higher scores. Specifically, significant differences were found in anxiety, depression, stress, and the total scale score. In terms of score interpretation, the mean scores among women corresponded to moderate levels of anxiety and depressive symptoms and mild levels of stress, whereas the mean scores among men corresponded to mild levels of anxiety and depressive symptoms and normal levels of stress.

For the SMRL, statistically significant differences were observed in idealized love, distorted love, and the total score, with men obtaining higher scores across all dimensions. These findings reflected greater endorsement of traditional romantic love myths among men, both for the total score and for the idealized and distorted love dimensions, whereas women showed lower endorsement, particularly for distorted love.

Regarding the Cib-VPA, statistically significant differences between men and women were found for cybercontrol victimization, cyberaggression victimization, cybercontrol perpetration, and total victimization, with women obtaining higher scores across all these dimensions. No significant differences were observed for cyberaggression perpetration or total perpetration. Nevertheless, the mean total victimization and total perpetration scores in both groups were below the theoretical midpoints of their respective ranges, suggesting an overall low frequency of digital dating violence behaviors in the sample. The magnitude of these between-sex differences varied, and some statistically significant differences were small, particularly for cybercontrol perpetration, indicating that statistical significance should not be interpreted as implying a large between-sex difference.

The partial Pearson correlation analyses, controlling for problematic social media use, are presented separately for men and women in Table 2. Among men, romantic love myths were positively and significantly associated with cybercontrol perpetration, cyberaggression perpetration, and total perpetration, whereas psychological distress was not significantly associated with any dimension of digital dating violence victimization or perpetration. Among women, romantic love myths were also positively associated with cyberaggression perpetration and total perpetration, whereas psychological distress was significantly associated with cybercontrol victimization, cybercontrol perpetration, and total victimization. Overall, these correlations suggest that romantic love myths were more consistently related to perpetration, whereas psychological distress showed a clearer association with victimization and cybercontrol among women.

Formal differences between men and women in the DASS-21 × SMRL interaction were examined using pooled regression models (Table 3). After Benjamini–Hochberg correction, the Sex × DASS-21 × SMRL interaction was statistically significant for victimization–cyberaggression (*B* = 0.00410, HC3 SE = 0.00145, 95% CI [0.00124, 0.00696], BH-adjusted *p* = 0.031), perpetration–cybercontrol (*B* = 0.00069, HC3 SE = 0.00030, 95% CI [0.00009, 0.00128], BH-adjusted *p* = 0.047), and total victimization (*B* = 0.00368, HC3 SE = 0.00161, 95% CI [0.00050, 0.00686], BH-adjusted *p* = 0.047). Because sex was coded as 0 = men and 1 = women, the positive coefficients indicated a more positive DASS-21 × SMRL interaction among women for these outcomes. No statistically significant sex differences in the interaction were found for victimization–cybercontrol, perpetration–cyberaggression, or total perpetration after correction for multiple comparisons. Thus, formal sex differences in the interaction were limited to three of the six digital dating violence outcomes examined.

Sex-stratified linear regression analyses were subsequently conducted to characterize, within each sex, the interaction between psychological distress and romantic love myths after controlling for problematic social media use (Table 4 and Table 5). DASS-21 and SMRL scores were mean-centered separately within each sex before computing the interaction term. Residual diagnostics indicated some departures from normality in certain models, and inspection of residual plots suggested some evidence of heteroscedasticity; therefore, statistical inference was based on HC3 robust standard errors. No influential cases were identified according to the conventional Cook’s distance threshold of 1. No problematic multicollinearity was observed based on VIF values. Among men, the interaction term was significant for cybercontrol victimization, cyberaggression perpetration, and total perpetration, even after applying the Benjamini–Hochberg correction. The full models in which the interaction was significant explained between 16.1% and 35.5% of the variance, with the interaction term accounting for an additional 8.2% to 11.3%. Among women, the interaction was significant for all the dimensions examined: cybercontrol victimization, cyberaggression victimization, cybercontrol perpetration, cyberaggression perpetration, total victimization, and total perpetration, even after applying the Benjamini–Hochberg correction. In this group, the full models explained between 6.4% and 24.4% of the variance, with the interaction term accounting for an additional 2.1% to 6.3%. These results indicate that the incremental contribution of the interaction was modest in several models, particularly among women, despite reaching statistical significance. Overall, significant within-group interactions were observed for several outcomes, but these patterns should be interpreted alongside the formal between-sex comparisons presented in Table 3.

**Figure 1:**
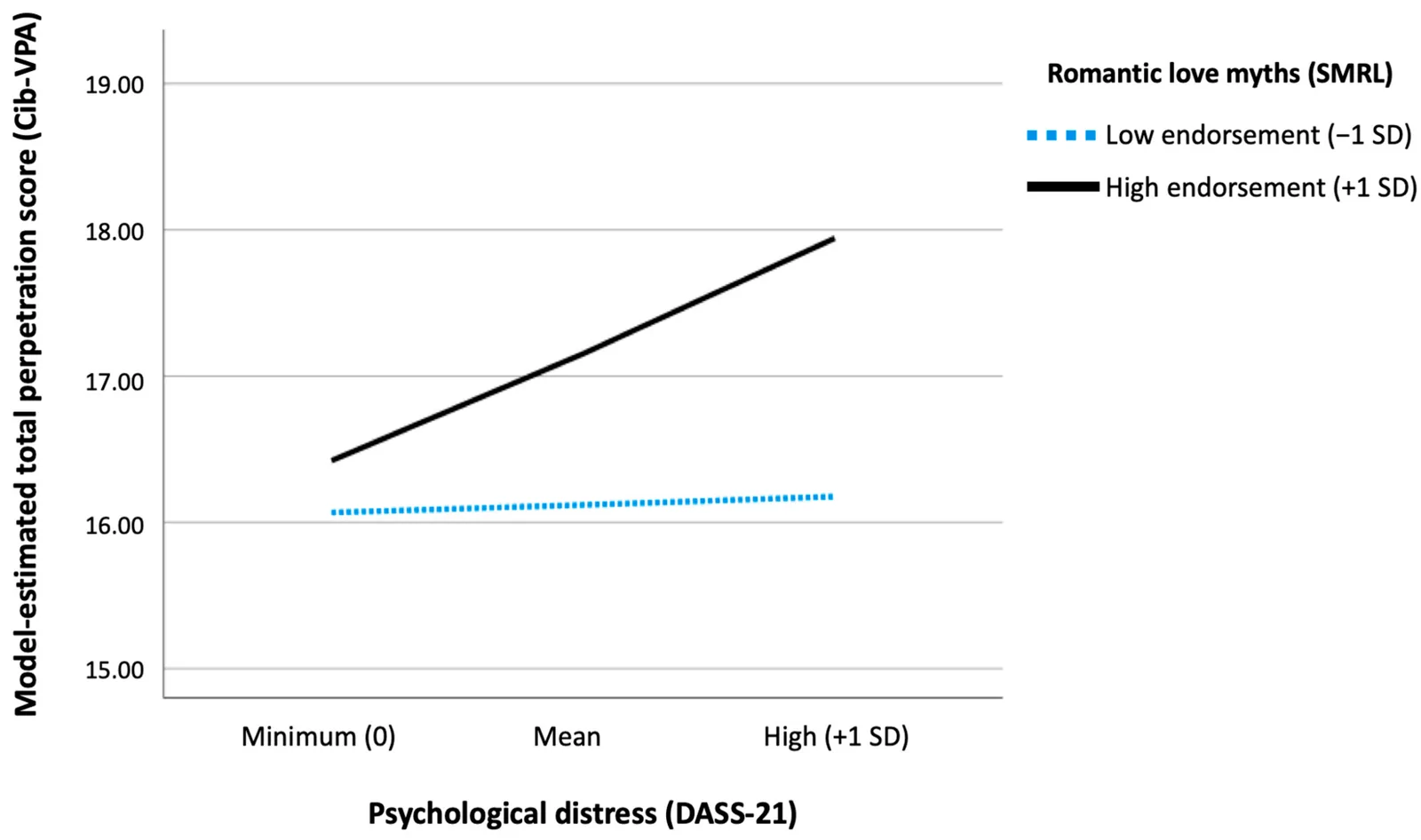
Interaction between psychological distress and romantic love myths in total perpetration among men. Note. Model-estimated total perpetration scores are presented at low (−1 SD) and high (+1 SD) levels of endorsement of romantic love myths. Psychological distress is represented at the theoretical minimum (0), the mean, and one standard deviation above the mean. Estimates were adjusted for problematic social media use.

**Figure 2:**
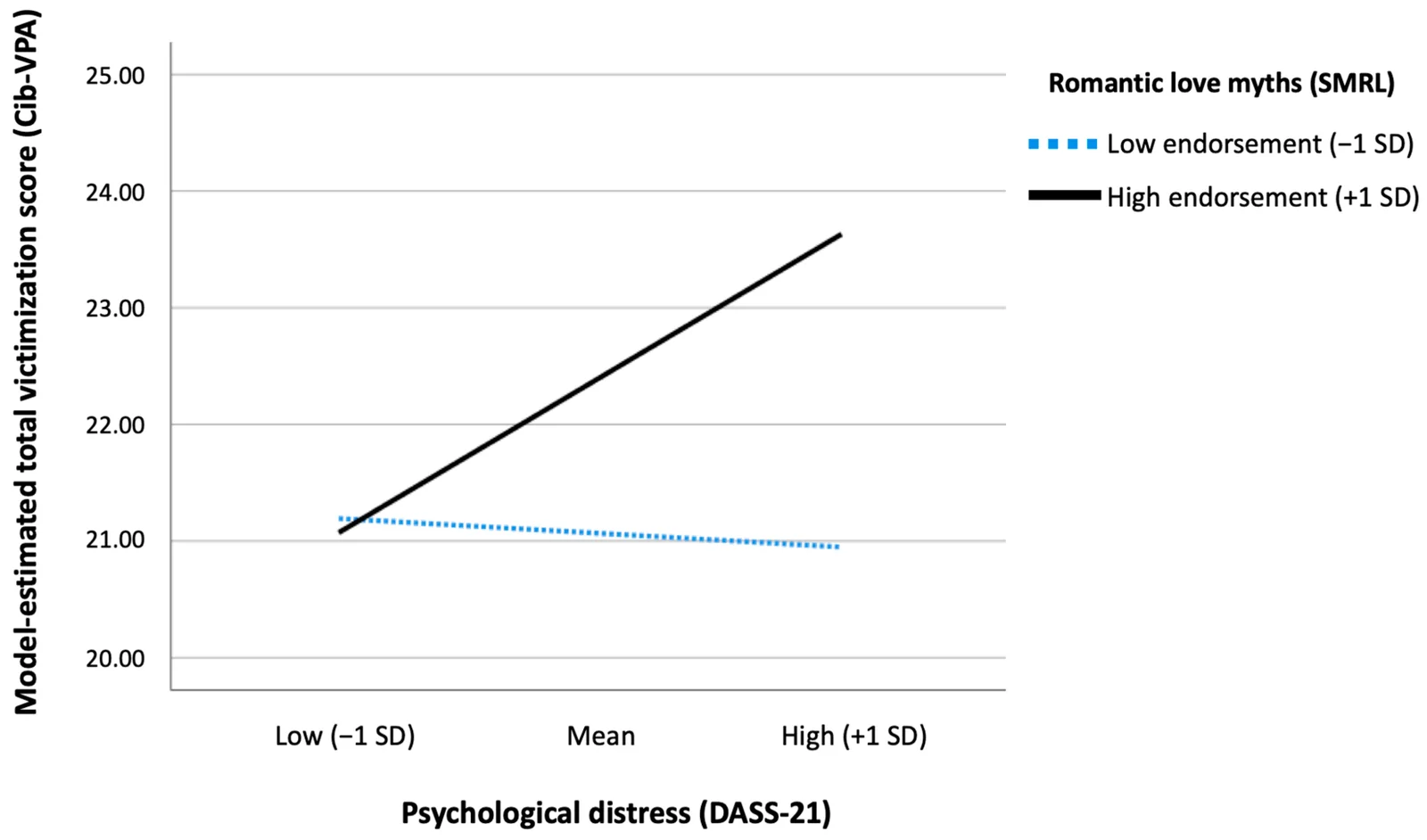
Interaction between psychological distress and romantic love myths in total victimization among women. Note. Model-estimated total victimization scores are presented at low (−1 SD) and high (+1 SD) levels of endorsement of romantic love myths. Psychological distress is represented at −1 SD, the mean, and +1 SD. Estimates were adjusted for problematic social media use.

**Figure 3:**
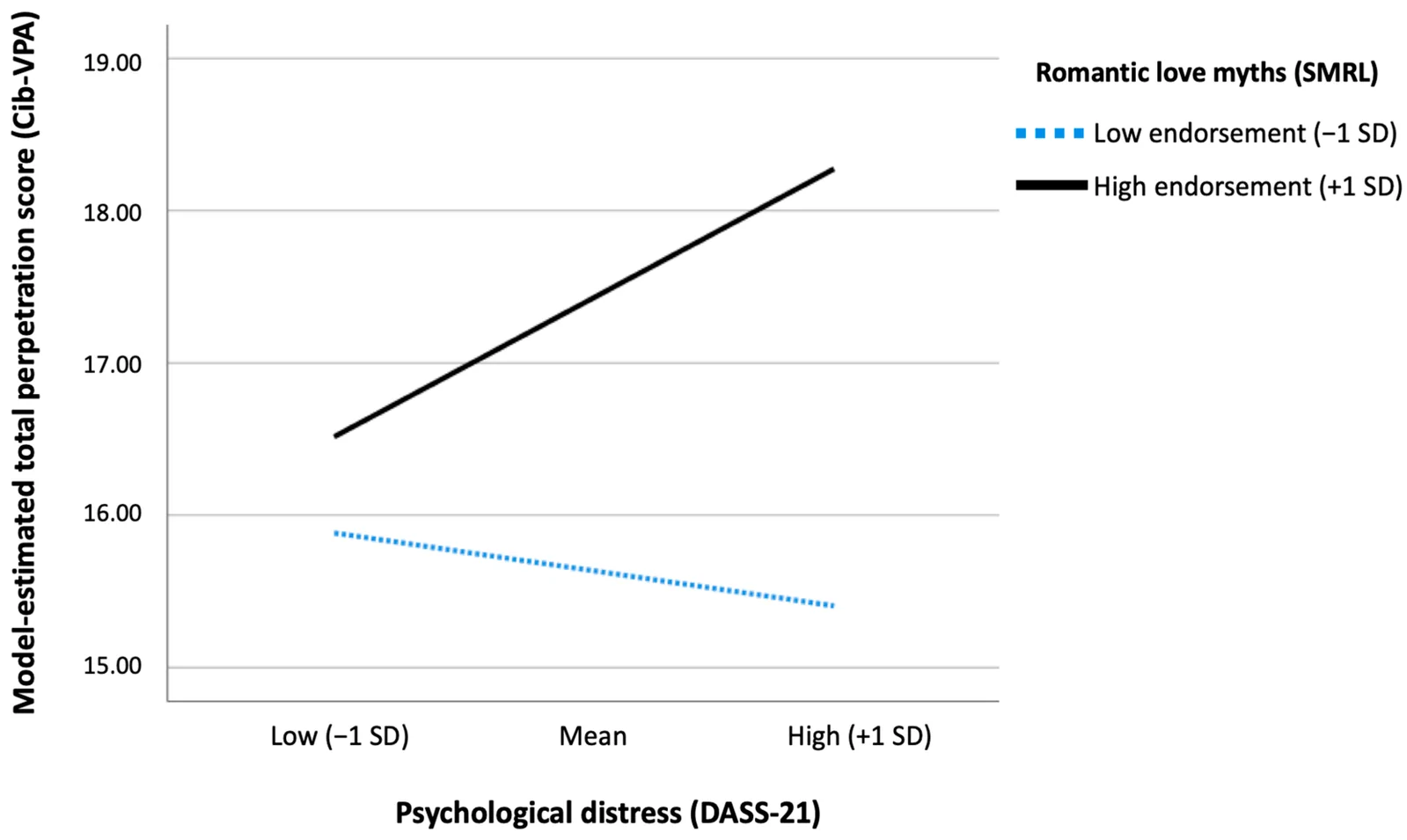
Interaction between psychological distress and romantic love myths in total perpetration among women. Note. Model-estimated total perpetration scores are presented at low (−1 SD) and high (+1 SD) levels of endorsement of romantic love myths. Psychological distress is represented at −1 SD, the mean, and +1 SD. Estimates were adjusted for problematic social media use.

To facilitate an overall interpretation of the interactions, simple slopes were examined in the models based on the total digital dating violence scores using HC3 standard errors. Specifically, these analyses were conducted for total perpetration among men and for total victimization and total perpetration among women, as these were the total scores for which the interaction between psychological distress and romantic love myths remained significant after the Benjamini–Hochberg correction. Among men, psychological distress was not significantly associated with total perpetration when endorsement of romantic love myths was low, *B* = 0.002, 95% CI [−0.008, 0.011], *p* = 0.735. However, when endorsement of these myths was high, psychological distress was positively associated with total perpetration, *B* = 0.023, 95% CI [0.014, 0.031], *p* < 0.001 (Figure 1). Among women, psychological distress was not significantly associated with total victimization when endorsement of romantic love myths was low, *B* = −0.004, 95% CI [−0.027, 0.019], *p* = 0.728. In contrast, when endorsement was high, psychological distress was positively associated with total victimization, *B* = 0.042, 95% CI [0.022, 0.062], *p* < 0.001 (Figure 2). The same pattern was observed for total perpetration: the association was not significant when endorsement of romantic love myths was low, *B* = −0.008, 95% CI [−0.026, 0.010], *p* = 0.378, but was positive and significant when endorsement was high, *B* = 0.029, 95% CI [0.015, 0.043], *p* < 0.001 (Figure 3).

## 4. Discussion

Romantic relationships among young people now develop within a context of high digital connectivity, in which dynamics of intimacy, communication, and control may extend into online environments. In this context, behaviors such as cybercontrol and cyberaggression are particularly relevant because they share characteristics with psychologically abusive and coercive-control dynamics within intimate relationships, including surveillance, monitoring, and restriction of a partner’s autonomy [1,2,3,6]. Recent studies have also documented associations and overlap between digital dating violence and offline forms of intimate partner violence [8,11,12]. However, research on the psychological and cognitive factors associated with these dynamics among young adults in romantic relationships remains limited, particularly when psychological distress, romantic love myths, and problematic social media use are examined jointly [7,14,21,22]. Against this background, the present study aimed to analyze the associations among psychological distress, romantic love myths, and digital dating violence among young adults in romantic relationships, considering both victimization and perpetration experiences.

The analysis of differences between men and women showed that women reported significantly higher levels of anxiety, depression, stress, and overall psychological distress. Their mean scores corresponded to moderate levels of anxiety and depressive symptoms and mild levels of stress, compared with the mild or normal levels observed among men. These findings are consistent with previous studies reporting a higher prevalence of anxiety, depression, and stress symptoms among young women, as well as greater vulnerability to emotional distress during emerging adulthood [45,46]. Regarding romantic love myths, men obtained significantly higher scores for idealized love, distorted love, and the overall endorsement of these beliefs, reflecting greater adherence to traditional romantic beliefs among men. This finding is consistent with Martínez-Soto et al. [22], who found that adolescent boys endorsed romantic love myths more strongly than girls and that these myths had mediating effects on digital dating violence. Similarly, Triano-López et al. [25] found that greater internalization of romantic love myths was associated with greater tolerance of cyber dating harassment. From a gender perspective, these patterns may be understood in relation to socialization processes and romantic culture, which continue to promote beliefs in which control and jealousy are interpreted as expressions of love [22,23]. Among young people, endorsement of romantic love myths has also been associated with a reduced ability to recognize certain abusive behaviors as forms of dating violence, particularly in digital contexts, where practices such as demanding passwords, monitoring social media activity, or requiring constant availability may be perceived as expressions of care or romantic interest [10,21].

Regarding digital dating violence, mean scores were generally low in both groups for victimization and perpetration, indicating a low average frequency of the behaviors assessed. This low frequency is important when interpreting the subsequent associations, particularly those involving perpetration, as restricted variability may reduce the precision with which associations can be estimated. Accordingly, significant findings involving perpetration should be interpreted within the context of a sample characterized by generally low levels of digital dating violence, rather than as evidence of frequent or severe perpetration. Nevertheless, significant differences between men and women were found for cybercontrol victimization, cyberaggression victimization, cybercontrol perpetration, and total victimization, with women obtaining higher scores. However, the magnitude of these differences varied, and some effects were small; therefore, they should not be interpreted as indicating large or uniform differences between men and women across dimensions. This finding is consistent with previous studies indicating that women may be more exposed to certain forms of digital victimization within romantic relationships, although the magnitude and direction of these differences may vary according to the specific behavior examined [7,13,16,47]. In addition, the observed differences in digital dating violence were not accompanied by between-group differences in problematic social media use and may also be considered in relation to broader relational and gender-related factors, including the normalization of control, jealousy, and digital surveillance in young relationships [18,22]. This normalization is particularly relevant within the framework of domestic violence, as coercive control is characterized by the accumulation of behaviors involving surveillance, restriction, intimidation, and regulation of a partner’s autonomy, which may now be transferred to digital environments through social media, instant messaging, geolocation, or device monitoring [1,3]. These between-sex differences should nevertheless be interpreted within the characteristics of the present sample, which consisted exclusively of students from a single Spanish university and included a higher proportion of women. Accordingly, they should not be assumed to generalize directly to non-university young adults or to other regional or sociocultural contexts.

The sex-stratified partial correlation analyses characterized the associations of psychological distress and romantic love myths with digital dating violence separately among men and women after controlling for problematic social media use. Among men, romantic love myths were associated with cybercontrol perpetration, cyberaggression perpetration, and total perpetration, whereas psychological distress was not significantly associated with any dimension. This pattern suggests that, among men, traditional romantic beliefs may be particularly linked to the perpetration of digital dating violence, in line with studies indicating that cognitive distortions about love may contribute to the tolerance or normalization of abusive behaviors in digital contexts [21,22,25]. Among women, romantic love myths were also associated with perpetration dimensions, specifically cyberaggression perpetration and total perpetration, whereas psychological distress was mainly associated with cybercontrol victimization, total victimization, and cybercontrol perpetration. This pattern suggests that romantic love myths were more consistently associated with the perpetration of digital dating violence, whereas psychological distress was more consistently associated with victimization and cybercontrol dynamics among women. This finding is consistent with studies reporting higher anxiety and depressive symptoms among young people who have experienced dating violence and greater psychological symptomatology among victims of online violence [14,15,48]. It may also be linked to evidence associating psychological distress with greater attachment insecurity, emotional dependence, and the use of digital surveillance as a maladaptive strategy for coping with fear of abandonment [18].

Formal pooled-model comparisons showed that the DASS-21 × SMRL interaction differed between men and women for cyberaggression victimization, cybercontrol perpetration, and total victimization after correction for multiple comparisons. In all three cases, the positive Sex × DASS-21 × SMRL coefficient indicated a more positive interaction among women. No formal sex difference was identified for cybercontrol victimization, cyberaggression perpetration, or total perpetration; therefore, differences in statistical significance between the corresponding sex-stratified models should not be interpreted as evidence that the associations differed by sex for these outcomes. The sex-stratified models nevertheless helped characterize the within-group patterns. Among men, the interaction was significant for cybercontrol victimization, cyberaggression perpetration, and total perpetration, whereas among women it was significant for all six outcomes. Although several interaction terms were statistically significant, their incremental contribution to explained variance was modest in several models, particularly among women. The simple slope analyses conducted for the total scores showed that psychological distress was positively associated with total perpetration among men when endorsement of romantic love myths was high, but not when it was low. The same pattern was found among women for both total victimization and total perpetration. These findings indicate that the association between psychological distress and some dimensions of digital dating violence was stronger at higher levels of romantic love myth endorsement, consistent with previous evidence [18,21].

From a theoretical perspective, these findings extend current accounts of digital dating violence by suggesting that cognitive and emotional factors may operate jointly rather than independently. Specifically, the pattern observed is consistent with a cognitive–emotional amplification perspective, whereby the association between psychological distress and digital dating violence is stronger at higher levels of romantic love myth endorsement. Nevertheless, given the cross-sectional design of the study, these findings do not allow the direction of the associations to be established or causal conclusions to be drawn.

This study has several limitations that should be considered when interpreting the findings. First, the cross-sectional design precludes causal inferences; therefore, the direction of the associations among psychological distress, romantic love myths, and digital dating violence should be interpreted with caution. Second, the greater representation of women may have affected the relative precision of the sex-specific estimates. Although between-sex differences in the focal interaction were formally tested using pooled models, the smaller sample of men may have reduced precision. The sex-stratified estimates should therefore be interpreted cautiously, particularly for outcomes for which the Sex × DASS-21 × SMRL interaction was not statistically significant. Third, the sample consisted exclusively of students from a single Spanish university, and recruitment was limited to the three participating faculties. These characteristics may limit the representativeness of the sample with respect to the wider university population and the generalizability of the findings to non-university young adults and to populations from other regions or sociocultural contexts. This limitation is particularly relevant for romantic love myths, as their endorsement and meaning may vary across cultural settings. Fourth, the assessment was based on self-report measures, which may introduce response biases, including social desirability, minimization of abusive behaviors, and subjective perceptions of the behaviors assessed. In addition, some conceptual proximity may exist between problematic social media use and cybercontrol, as both involve behaviors occurring in digital environments. However, they represent distinct constructs, with problematic social media use referring to maladaptive patterns of social media engagement and cybercontrol referring specifically to controlling behaviors directed toward a romantic partner. Furthermore, the overall low frequency of digital dating violence in the sample may have reduced variability in some dimensions, particularly the perpetration scales, thereby limiting the detection and precision of associations. This also warrants caution when considering the clinical significance of the perpetration findings, as the present results reflect associations within a predominantly low-frequency sample rather than clinically defined or severe patterns of abusive behavior.

Despite these limitations, the present study contributes to the growing body of evidence on digital dating violence among young adults in romantic relationships by jointly examining cognitive, emotional, and technological factors. Controlling for problematic social media use also made it possible to examine the associations of psychological distress and romantic love myths with digital dating violence beyond the maladaptive use of these platforms. An additional strength is the distinction between victimization and perpetration and between cybercontrol and cyberaggression, allowing for a more precise examination of different forms of involvement in digital dating violence. Overall, the findings are consistent with previous evidence showing associations between digital dating violence, psychological abuse, and other forms of intimate partner violence [3,8,12,49,50]. From an applied perspective, these findings reinforce the need to include digital manifestations of control and aggression in intimate partner violence prevention programs for young people [2,8,22]. Such programs could include psychoeducational activities to improve recognition of digital surveillance, demands for passwords, geolocation monitoring, or expectations of constant availability as potentially abusive behaviors; cognitive components aimed at challenging beliefs that equate jealousy, possessiveness, or control with love; and emotional regulation and adaptive coping strategies for managing psychological distress within romantic relationships. Given the generally low frequency of these behaviors in the present sample, these implications are most appropriately framed in terms of prevention and recognition of potentially abusive digital behaviors rather than clinical risk identification. Future research should use more balanced samples and longitudinal designs to examine the temporal relationships between digital dating violence and offline forms of psychological violence, coercive control, or domestic violence in adult relationships.

## 5. Conclusions

The present study provides empirical evidence regarding the multidimensional nature of digital dating violence among young adults in romantic relationships. The findings indicate that digital dating violence was associated with emotional and cognitive factors, including psychological distress and romantic love myths. More specifically, psychological distress was mainly associated with victimization experiences and cybercontrol dynamics, whereas romantic love myths were primarily associated with the perpetration of digital controlling or aggressive behaviors. The simple slope analyses further showed that the association between psychological distress and total perpetration among men and women, and between psychological distress and total victimization among women, was positive when endorsement of romantic love myths was high but was not significant when endorsement was low. From this perspective, cybercontrol, cyberaggression, and stronger endorsement of romantic love myths may be considered in relation to psychologically abusive and coercive dynamics in intimate relationships. Previous research has also documented associations between digital dating violence and other forms of intimate partner violence, although the present cross-sectional findings cannot establish temporal continuity between them. Accordingly, intimate partner violence prevention programs may benefit from simultaneously challenging romantic love myths, improving the recognition of cybercontrol as a form of violence, and supporting psychological well-being and adaptive coping among young people, while acknowledging the digital environment as an important context in which both healthy and abusive relationship dynamics may occur.

## Figures and Tables

**Table 1:** Comparison of men and women on the BSMAS, DASS-21, SMRL, and Cib-VPA.

	Men (*n* = 124)	Women (*n* = 212)	Total (*n* = 336)	*t* (df)	Hedges’ *g* [95% CI]	Adjusted *p*-Value
BSMAS	16.27 ± 4.09	17.30 ± 4.98	16.92 ± 4.69	1.951 (334)	0.220 [−0.002, 0.442]	0.061
DASS-21						
Anxiety	8.73 ± 12.39	13.53 ± 11.55	11.76 ± 12.07	3.580 (334)	0.404 [0.180, 0.628]	<0.001
Depression	10.65 ± 12.65	14.69 ± 11.64	13.20 ± 12.16	2.975 (334)	0.336 [0.113, 0.559]	0.004
Stress	12.95 ± 10.92	17.85 ± 10.12	16.04 ± 10.67	4.156 (334)	0.469 [0.244, 0.693]	<0.001
Total score	32.32 ± 34.91	46.07 ± 30.33	40.99 ± 32.73	3.788 (334)	0.427 [0.203, 0.651]	<0.001
SMRL						
Idealized love	21.46 ± 4.83	18.96 ± 5.61	19.88 ± 5.46	−4.316 (288.866)	−0.468 [−0.693, −0.244]	<0.001
Distorted love	11.26 ± 3.57	9.04 ± 2.77	9.86 ± 3.27	−5.953 (209.644)	−0.717 [−0.945, −0.489]	<0.001
Total score	32.72 ± 7.16	28.00 ± 7.24	29.74 ± 7.55	−5.793 (334)	−0.653 [−0.881, −0.426]	<0.001
Cib-VPA						
Victimization–Cybercontrol	8.90 ± 2.28	11.37 ± 2.75	10.46 ± 2.84	8.851 (296.376)	0.951 [0.718, 1.184]	<0.001
Victimization–Cyberaggression	9.19 ± 3.35	10.33 ± 1.58	9.90 ± 2.45	3.561 (155.610)	0.475 [0.251, 0.700]	<0.001
Perpetration–Cybercontrol	9.96 ± 0.60	10.12 ± 0.57	10.06 ± 0.59	2.408 (334)	0.272 [0.049, 0.494]	0.021
Perpetration–Cyberaggression	6.65 ± 1.29	6.39 ± 2.49	6.49 ± 2.13	−1.288 (330.125)	−0.125 [−0.347, 0.097]	0.214
Total victimization	18.09 ± 4.73	21.69 ± 3.56	20.36 ± 4.39	7.351 (204.751)	0.892 [0.660, 1.124]	<0.001
Total perpetration	16.61 ± 1.45	16.50 ± 2.76	16.54 ± 2.36	−0.470 (330.794)	−0.046 [−0.267, 0.176]	0.639

Note. BSMAS = Bergen Social Media Addiction Scale; DASS-21 = Depression Anxiety Stress Scales-21; SMRL = Scale of Myths of Romantic Love; Cib-VPA = Cyber-Violence in Adolescent Couples Scale; CI = confidence interval. Descriptive values are presented as mean ± standard deviation. Effect sizes are expressed as Hedges’ *g* with 95% confidence intervals. Positive values indicate higher scores among women, whereas negative values indicate higher scores among men. The *p*-values were adjusted using the Benjamini–Hochberg procedure across the 14 comparisons reported in the table.

**Table 2:** Partial correlations of psychological distress and romantic love myths with digital dating violence, controlling for problematic social media use.

	Men (*n* = 124)		Women (*n* = 212)	
	DASS-21	SMRL	DASS-21	SMRL
Cib-VPA				
Victimization–Cybercontrol	0.208	0.166	0.179 *	0.087
Victimization–Cyberaggression	−0.023	0.114	0.071	0.138
Perpetration–Cybercontrol	0.170	0.250 *	0.190 *	0.119
Perpetration–Cyberaggression	0.172	0.380 **	0.084	0.271 **
Total victimization	0.084	0.161	0.169 *	0.124
Total perpetration	0.224	0.442 **	0.115	0.267 **

Note. DASS-21 = Depression Anxiety Stress Scales-21; SMRL = Scale of Myths of Romantic Love; Cib-VPA = Cyber-Violence in Adolescent Couples Scale. The *p*-values were obtained from partial Pearson correlation analyses controlling for problematic social media use and were adjusted using the Benjamini–Hochberg procedure separately across the six digital dating violence outcomes for each predictor within each sex. * *p* < 0.05; ** *p* < 0.01.

**Table 3:** Pooled linear regression models testing sex differences in the interaction between psychological distress and romantic love myths across digital dating violence outcomes.

	*B*	HC3 SE	95% CI for *B*	*p*	BH-Adjusted *p*
Victimization–Cybercontrol	−0.00042	0.00102	[−0.00243, 0.00159]	0.683	0.683
Victimization–Cyberaggression	0.00410	0.00145	[0.00124, 0.00696]	0.005	0.031
Perpetration–Cybercontrol	0.00069	0.00030	[0.00009, 0.00128]	0.023	0.047
Perpetration–Cyberaggression	0.00040	0.00075	[−0.00107, 0.00187]	0.594	0.683
Total victimization	0.00368	0.00161	[0.00050, 0.00686]	0.023	0.047
Total perpetration	0.00108	0.00086	[−0.00061, 0.00278]	0.209	0.314

Note. HC3 SE = heteroscedasticity-consistent standard error (HC3); CI = confidence interval; BSMAS = Bergen Social Media Addiction Scale; DASS-21 = Depression Anxiety Stress Scales-21; SMRL = Scale of Myths of Romantic Love; BH = Benjamini–Hochberg. *B* represents the coefficient for the Sex × DASS-21 × SMRL interaction. Sex was coded as 0 = men and 1 = women; therefore, positive coefficients indicate a more positive DASS-21 × SMRL interaction among women than among men. DASS-21 and SMRL scores were mean-centered within sex before calculating the interaction terms. Models included BSMAS, Sex, DASS-21, SMRL, all lower-order terms involved in the Sex × DASS-21 × SMRL interaction, and the Sex × BSMAS interaction. Statistical inference was based on HC3 heteroscedasticity-consistent standard errors. The *p*-values shown are two-tailed and unadjusted. Benjamini–Hochberg-adjusted *p*-values were calculated across the six Sex × DASS-21 × SMRL interaction tests.

**Table 4:** Linear regression models examining the interaction between psychological distress and romantic love myths in digital dating violence among men, controlling for problematic social media use.

Men (*n* = 124)								
Dependent Variable	Model Fit	Predictor	*B*	HC3 SE	β	95% CI for *B*	*p*	VIF
Victimization–Cybercontrol	*R*^2^ = 0.161; Adjusted *R*^2^ = 0.133; Δ*R*^2^ = 0.082, 95% CI [0.020, 0.171]; HC3 *F*(4, 119) = 9.912; *p* < 0.001	BSMAS	−0.059	0.050	−0.106	[−0.158, 0.040]	0.241	1.195
		DASS-21	0.016	0.006	0.242	[0.004, 0.028]	0.011	1.192
		SMRL	0.024	0.025	0.076	[−0.024, 0.073]	0.328	1.177
		DASS-21 × SMRL	0.00226	0.00056	0.309	[0.00115, 0.00336]	<0.001	1.157
Victimization–Cyberaggression	*R*^2^ = 0.071; Adjusted *R*^2^ = 0.040; Δ*R*^2^ = 0.056, 95% CI [0.000, 0.178]; HC3 *F*(4, 119) = 1.758; *p* = 0.142	BSMAS	0.029	0.087	0.035	[−0.143, 0.201]	0.739	1.195
		DASS-21	−0.001	0.011	−0.008	[−0.023, 0.021]	0.945	1.192
		SMRL	0.097	0.051	0.208	[−0.003, 0.198]	0.057	1.177
		DASS-21 × SMRL	−0.00274	0.00141	−0.255	[−0.00554, 0.00005]	0.054	1.157
Perpetration–Cybercontrol	*R*^2^ = 0.101; Adjusted *R*^2^ = 0.071; Δ*R*^2^ < 0.001, 95% CI [0.000, 0.032]; HC3 *F*(4, 119) = 2.779; *p* = 0.030	BSMAS	−0.015	0.016	−0.099	[−0.045, 0.016]	0.352	1.195
		DASS-21	0.004	0.002	0.216	[0.0003, 0.0072]	0.035	1.192
		SMRL	0.023	0.008	0.277	[0.008, 0.039]	0.004	1.177
		DASS-21 × SMRL	−0.00003	0.00018	−0.016	[−0.00038, 0.00032]	0.864	1.157
Perpetration–Cyberaggression	*R*^2^ = 0.302; Adjusted *R*^2^ = 0.279; Δ*R*^2^ = 0.113, 95% CI [0.017, 0.217]; HC3 *F*(4, 119) = 10.449; *p* < 0.001	BSMAS	−0.037	0.028	−0.116	[−0.092, 0.019]	0.192	1.195
		DASS-21	0.008	0.003	0.226	[0.002, 0.015]	0.010	1.192
		SMRL	0.049	0.015	0.270	[0.020, 0.078]	0.001	1.177
		DASS-21 × SMRL	0.00150	0.00037	0.361	[0.00076, 0.00224]	<0.001	1.157
Total victimization	*R*^2^ = 0.038; Adjusted *R*^2^ = 0.006; Δ*R*^2^ = 0.001, 95% CI [0.000, 0.027]; HC3 *F*(4, 119) = 1.505; *p* = 0.205	BSMAS	−0.030	0.122	−0.026	[−0.272, 0.212]	0.806	1.195
		DASS-21	0.015	0.014	0.111	[−0.012, 0.042]	0.276	1.192
		SMRL	0.121	0.059	0.184	[0.005, 0.238]	0.041	1.177
		DASS-21 × SMRL	−0.00049	0.00125	−0.032	[−0.00297, 0.00199]	0.698	1.157
Total perpetration	*R*^2^ = 0.355; Adjusted *R*^2^ = 0.334; Δ*R*^2^ = 0.086, 95% CI [0.012, 0.175]; HC3 *F*(4, 119) = 13.695; *p* < 0.001	BSMAS	−0.051	0.031	−0.144	[−0.113, 0.010]	0.102	1.195
		DASS-21	0.012	0.003	0.291	[0.005, 0.019]	<0.001	1.192
		SMRL	0.072	0.015	0.356	[0.042, 0.102]	<0.001	1.177
		DASS-21 × SMRL	0.00147	0.00040	0.315	[0.00068, 0.00226]	<0.001	1.157

Note. HC3 SE = heteroscedasticity-consistent standard error (HC3); CI = confidence interval; VIF = variance inflation factor. DASS-21 and SMRL scores were mean-centered separately within each sex before calculating the interaction term. All models were adjusted for BSMAS scores. *R*^2^ and adjusted *R*^2^ correspond to the full ordinary least-squares model. Δ*R*^2^ represents the increase in *R*^2^ obtained by adding the DASS-21 × SMRL interaction term to a reduced model containing BSMAS and the two mean-centered main effects and is reported as an index of the incremental magnitude of the interaction effect. The precision of the focal interaction effects is represented by the corresponding 95% confidence intervals for *B* calculated using HC3 standard errors. HC3 *F* represents the robust Wald test of overall model significance. The *p*-values shown for the individual predictors are two-tailed and unadjusted. The Benjamini–Hochberg correction was applied separately within each sex across the six DASS-21 × SMRL interaction tests. Benjamini–Hochberg-adjusted *p*-values for the DASS-21 × SMRL interaction were as follows: Victimization–Cybercontrol, <0.001; Victimization–Cyberaggression, 0.081; Perpetration–Cybercontrol, 0.864; Perpetration–Cyberaggression, <0.001; Total victimization, 0.838; and Total perpetration, <0.001.

**Table 5:** Linear regression models examining the interaction between psychological distress and romantic love myths in digital dating violence among women, controlling for problematic social media use.

Women (*n* = 212)								
Dependent Variable	Model Fit	Predictor	*B*	HC3 SE	β	95% CI for *B*	*p*	VIF
Victimization–Cybercontrol	*R*^2^ = 0.064; Adjusted *R*^2^ = 0.046; Δ*R*^2^ = 0.021, 95% CI [0.0003, 0.072]; HC3 *F*(4, 207) = 3.554; *p* = 0.008	BSMAS	−0.020	0.037	−0.035	[−0.093, 0.054]	0.600	1.101
		DASS-21	0.016	0.006	0.178	[0.004, 0.028]	0.010	1.079
		SMRL	0.051	0.029	0.134	[−0.005, 0.107]	0.076	1.082
		DASS-21 × SMRL	0.00184	0.00086	0.150	[0.00015, 0.00353]	0.033	1.058
Victimization–Cyberaggression	*R*^2^ = 0.244; Adjusted *R*^2^ = 0.229; Δ*R*^2^ = 0.035, 95% CI [0.007, 0.075]; HC3 *F*(4, 207) = 18.311; *p* < 0.001	BSMAS	−0.150	0.019	−0.471	[−0.187, −0.112]	<0.001	1.101
		DASS-21	0.003	0.003	0.057	[−0.004, 0.009]	0.369	1.079
		SMRL	0.038	0.012	0.172	[0.013, 0.062]	0.003	1.082
		DASS-21 × SMRL	0.00135	0.00035	0.192	[0.00066, 0.00204]	<0.001	1.058
Perpetration–Cybercontrol	*R*^2^ = 0.117; Adjusted *R*^2^ = 0.100; Δ*R*^2^ = 0.063, 95% CI [0.002, 0.162]; HC3 *F*(4, 207) = 3.774; *p* = 0.006	BSMAS	−0.008	0.008	−0.070	[−0.024, 0.008]	0.325	1.101
		DASS-21	0.003	0.002	0.181	[0.0003, 0.0065]	0.031	1.079
		SMRL	0.015	0.007	0.190	[0.002, 0.028]	0.024	1.082
		DASS-21 × SMRL	0.00066	0.00024	0.258	[0.00017, 0.00114]	0.008	1.058
Perpetration–Cyberaggression	*R*^2^ = 0.176; Adjusted *R*^2^ = 0.160; Δ*R*^2^ = 0.027, 95% CI [0.003, 0.074]; HC3 *F*(4, 207) = 13.185; *p* < 0.001	BSMAS	−0.167	0.030	−0.334	[−0.226, −0.108]	<0.001	1.101
		DASS-21	0.007	0.005	0.087	[−0.003, 0.018]	0.178	1.079
		SMRL	0.106	0.022	0.308	[0.063, 0.149]	<0.001	1.082
		DASS-21 × SMRL	0.00190	0.00065	0.171	[0.00062, 0.00317]	0.004	1.058
Total victimization	*R*^2^ = 0.114; Adjusted *R*^2^ = 0.097; Δ*R*^2^ = 0.038, 95% CI [0.004, 0.096]; HC3 *F*(4, 207) = 7.141; *p* < 0.001	BSMAS	−0.169	0.045	−0.237	[−0.259, −0.079]	<0.001	1.101
		DASS-21	0.019	0.008	0.163	[0.003, 0.035]	0.019	1.079
		SMRL	0.088	0.034	0.180	[0.022, 0.155]	0.010	1.082
		DASS-21 × SMRL	0.00319	0.00102	0.201	[0.00119, 0.00520]	0.002	1.058
Total perpetration	*R*^2^ = 0.183; Adjusted *R*^2^ = 0.167; Δ*R*^2^ = 0.040, 95% CI [0.005, 0.098]; HC3 *F*(4, 207) = 13.749; *p* < 0.001	BSMAS	−0.175	0.033	−0.316	[−0.241, −0.109]	<0.001	1.101
		DASS-21	0.011	0.006	0.116	[−0.001, 0.022]	0.081	1.079
		SMRL	0.121	0.025	0.317	[0.071, 0.171]	<0.001	1.082
		DASS-21 × SMRL	0.00255	0.00076	0.207	[0.00105, 0.00405]	<0.001	1.058

Note. HC3 SE = heteroscedasticity-consistent standard error (HC3); CI = confidence interval; VIF = variance inflation factor. DASS-21 and SMRL scores were mean-centered separately within each sex before calculating the interaction term. All models were adjusted for BSMAS scores. *R*^2^ and adjusted *R*^2^ correspond to the full ordinary least-squares model. Δ*R*^2^ represents the increase in *R*^2^ obtained by adding the DASS-21 × SMRL interaction term to a reduced model containing BSMAS and the two mean-centered main effects and is reported as an index of the incremental magnitude of the interaction effect. The precision of the focal interaction effects is represented by the corresponding 95% confidence intervals for *B* calculated using HC3 standard errors. HC3 *F* represents the robust Wald test of overall model significance. The *p*-values shown for the individual predictors are two-tailed and unadjusted. The Benjamini–Hochberg correction was applied separately within each sex across the six DASS-21 × SMRL interaction tests. Benjamini–Hochberg-adjusted *p*-values for the DASS-21 × SMRL interaction were as follows: Victimization–Cybercontrol, 0.033; Victimization–Cyberaggression, <0.001; Perpetration–Cybercontrol, 0.009; Perpetration–Cyberaggression, 0.006; Total victimization, 0.004; and Total perpetration, 0.003.

## Data Availability

The data presented in this study are available on request from the corresponding author. The data are not publicly available due to privacy reasons and ethical restrictions.

## Abbreviations

The following abbreviations are used in this manuscript:

BSMAS	Bergen Social Media Addiction Scale
Cib-VPA	Cyber-Violence in Adolescent Couples Scale
DASS-21	Depression Anxiety Stress Scales-21
SMRL	Scale of Myths of Romantic Love

## References

[1] Dragiewicz M., Burgess J., Matamoros-Fernández A., Salter M., Suzor N.P., Woodlock D., Harris B. (2018). Technology Facilitated Coercive Control: Domestic Violence and the Competing Roles of Digital Media Platforms. Fem. Media Stud..

[2] Henry N., Flynn A., Powell A. (2020). Technology-Facilitated Domestic and Sexual Violence: A Review. Violence Against Women.

[3] Woodlock D. (2017). The Abuse of Technology in Domestic Violence and Stalking. Violence Against Women.

[4] Dichter M.E., Thomas K.A., Crits-Christoph P., Ogden S.N., Rhodes K.V. (2018). Coercive Control in Intimate Partner Violence: Relationship with Women’s Experience of Violence, Use of Violence, and Danger. Psychol. Violence.

[5] Stark E., Hester M. (2019). Coercive Control: Update and Review. Violence Against Women.

[6] World Health Organization Violence Against Women. https://www.who.int/news-room/fact-sheets/detail/violence-against-women.

[7] Caridade S., Braga T., Borrajo E. (2019). Cyber Dating Abuse (CDA): Evidence from a Systematic Review. Aggress. Violent Behav..

[8] Rodríguez-deArriba M.-L., Santos C., Cunha O., Sánchez-Jiménez V., Caridade S. (2024). Relationship Between Cyber and In-Person Dating Abuse: A Systematic Review. Aggress. Violent Behav..

[9] Borrajo E., Gámez-Guadix M., Pereda N., Calvete E. (2015). The Development and Validation of the Cyber Dating Abuse Questionnaire among Young Couples. Comput. Hum. Behav..

[10] Machado B., de Faria P.L., Araújo I., Caridade S. (2024). Cyber Interpersonal Violence: Adolescent Perspectives and Digital Practices. Int. J. Environ. Res. Public Health.

[11] Lara L. (2020). Cyber Dating Abuse: Assessment, Prevalence, and Relationship with Offline Violence in Young Chileans. J. Soc. Pers. Relatsh..

[12] Lu Y., Van Ouytsel J., Temple J.R. (2021). In-Person and Cyber Dating Abuse: A Longitudinal Investigation. J. Soc. Pers. Relatsh..

[13] Zweig J.M., Dank M., Yahner J., Lachman P. (2013). The Rate of Cyber Dating Abuse among Teens and How It Relates to Other Forms of Teen Dating Violence. J. Youth Adolesc..

[14] Borrajo E., Gámez-Guadix M. (2016). Abuso “Online” En El Noviazgo: Relación Con Depresión, Ansiedad y Ajuste Diádico. Behav. Psychol..

[15] Jaureguizar J., Dosil-Santamaria M., Redondo I., Wachs S., Machimbarrena J.M. (2024). Online and Offline Dating Violence: Same Same, but Different?. Psicol. Reflexão Crít..

[16] Reed L.A., Tolman R.M., Ward L.M. (2017). Gender Matters: Experiences and Consequences of Digital Dating Abuse Victimization in Adolescent Dating Relationships. J. Adolesc..

[17] Zweig J.M., Lachman P., Yahner J., Dank M. (2014). Correlates of Cyber Dating Abuse among Teens. J. Youth Adolesc..

[18] García-Valcárcel M., Moral-Jiménez M.D.L.V. (2024). Dependencia emocional, ciberviolencia y control a través de las redes sociales en relaciones de pareja entre personas jóvenes. Actual. Psicol..

[19] Andreassen C.S., Billieux J., Griffiths M.D., Kuss D.J., Demetrovics Z., Mazzoni E., Pallesen S. (2016). The Relationship Between Addictive Use of Social Media and Video Games and Symptoms of Psychiatric Disorders: A Large-Scale Cross-Sectional Study. Psychol. Addict. Behav..

[20] Muñiz-Rivas M., Suárez-Relinque C., Estévez E., Povedano-Díaz A. (2023). Victims of Dating Violence in Adolescence: The Role of Problematic Use of Social Networks Sites, Loneliness, and Family Climate. Ann. Psychol..

[21] Borrajo E., Gámez-Guadix M., Calvete E. (2015). Justification Beliefs of Violence, Myths About Love and Cyber Dating Abuse. Psicothema.

[22] Martínez-Soto A., Lopez-del Burgo C., Albertos A., Ibabe I. (2024). Cyber Dating Abuse in Adolescents: Myths of Romantic Love, Sexting Practices and Bullying. Comput. Hum. Behav..

[23] Bonilla-Algovia E., Rivas-Rivero E., Pascual-Gómez I. (2021). Mitos del amor romántico en adolescentes: Relación con el sexismo y variables procedentes de la socialización. Educ. XX1.

[24] Ramírez-Carrasco D., Ferrer-Urbina R., Ponce-Correa F. (2023). Jealousy, Sexism, and Romantic Love Myths: The Role of Beliefs in Online Dating Violence. Front. Psychol..

[25] Triano-López P., Morales-Marente E., Palacios-Gálvez M.S. (2021). Tolerancia hacia el ciberacoso en el noviazgo: Analizando su relación con la violencia de género. HAAJ.

[26] Cava M.-J., Martínez-Ferrer B., Buelga S., Carrascosa L. (2020). Sexist Attitudes, Romantic Myths, and Offline Dating Violence as Predictors of Cyber Dating Violence Perpetration in Adolescents. Comput. Hum. Behav..

[27] Cava M.J., Buelga S., Carrascosa L., Ortega-Barón J. (2020). Relations among Romantic Myths, Offline Dating Violence Victimization and Cyber Dating Violence Victimization in Adolescents. Int. J. Environ. Res. Public Health.

[28] Rodríguez-deArriba M.-L., Nocentini A., Menesini E., Sánchez-Jiménez V. (2021). Dimensions and Measures of Cyber Dating Violence in Adolescents: A Systematic Review. Aggress. Violent Behav..

[29] von Elm E., Altman D.G., Egger M., Pocock S.J., Gøtzsche P.C., Vandenbroucke J.P. (2008). Strengthening the Reporting of Observational Studies in Epidemiology (STROBE) Statement: Guidelines for Reporting Observational Studies. J. Clin. Epidemiol..

[30] Hochberg Z., Konner M. (2020). Emerging Adulthood, a Pre-Adult Life-History Stage. Front. Endocrinol..

[31] Rauer A.J., Pettit G.S., Lansford J.E., Bates J.E., Dodge K.A. (2013). Romantic Relationship Patterns in Young Adulthood and Their Developmental Antecedents. Dev. Psychol..

[32] Barrera-Mesa C.E., Caro-Caro E.O., Rey-Alamillo R.D. (2022). Víctimas de ciberviolencia: Formas, prevalencia y diferencias de género. Rev. Investig. Desarro. Innov..

[33] De los Reyes V. (2025). Ciberviolencia en parejas adolescentes jóvenes: Una revisión. Rev. INFAD Psicol. Int. J. Dev. Educ. Psychol..

[34] Sisí C., Pallesen S., Fernández-Martín M.P. (2025). The Spanish Version of the Bergen Social Media Addiction Scale (BSMAS): Validity, Reliability and Measurement Invariance Across Gender in a Sample of Young Adults. Psychiatr. Q..

[35] Lovibond S.H., Lovibond P.F. (1995). Manual for the Depression Anxiety Stress Scales.

[36] Bados A., Solanas A., Andrés R. (2005). Psychometric Properties of the Spanish Version of Depression, Anxiety and Stress Scales (DASS). Psicothema.

[37] Fonseca-Pedrero E., de las Paino-Piñeiro M.M., Lemos-Giráldez S., Muñiz-Fernández J. (2010). Propiedades psicométricas de la Depression Anxiety and Stress Scales-21 (DASS-21) en universitarios españoles. Ansiedad Estrés.

[38] Bonilla-Algovia E., Rivas-Rivero E. (2020). Diseño y Validación de la Escala de Mitos del Amor Romántico. Rev. Iberoam. Diagnóstico Evaluación Avaliação Psicológica.

[39] Cava M.J., Buelga S. (2018). Propiedades psicométricas de la Escala de Ciber-Violencia en Parejas Adolescentes (Cib-VPA). Suma Psicológica.

[40] Redondo I., Jaureguizar J., Dosil M., Galende N. (2022). Measuring Cyber Dating Violence: Reliability and Validity of the Escala de CiberViolencia En Parejas Adolescentes (Cib-VPA) in Spanish Young Adults. Clín. Salud.

[41] Algina J., Keselman H.J., Penfield R.J. (2008). Note on a Confidence Interval for the Squared Semipartial Correlation Coefficient. Educ. Psychol. Meas..

[42] Benjamini Y., Hochberg Y. (1995). Controlling the False Discovery Rate: A Practical and Powerful Approach to Multiple Testing. J. R. Stat. Soc. Ser. B Methodol..

[43] Cohen J. (1988). Statistical Power Analysis for the Behavioral Sciences.

[44] Rodríguez-deArriba M.-L., Muñoz-Fernández N., Sánchez-Jiménez V. (2022). Stability of Cyber Dating Victimization and Psychological Adjustment in Adolescents: A Short-Term Longitudinal Study. Psychol. Soc. Educ..

[45] Farhane-Medina N.Z., Luque B., Tabernero C., Castillo-Mayén R. (2022). Factors Associated with Gender and Sex Differences in Anxiety Prevalence and Comorbidity: A Systematic Review. Sci. Prog..

[46] Ramón-Arbués E., Gea-Caballero V., Granada-López J.M., Juárez-Vela R., Pellicer-García B., Antón-Solanas I. (2020). The Prevalence of Depression, Anxiety and Stress and Their Associated Factors in College Students. Int. J. Environ. Res. Public Health.

[47] Sousa J., Rodríguez-Ávila N., Rodríguez-Martínez P. (2024). Ciberviolencia en España: Tipos, víctimas y agresores. Rev. Comun. SEECI.

[48] Tarriño-Concejero L., de los Ángeles García-Carpintero-Muñoz M., Barrientos-Trigo S., Gil-García E. (2023). Violencia En El Noviazgo y Su Relación Con La Ansiedad, La Depresión y El Estrés En Jóvenes Universitarios Andaluces. Enfermería Clínica.

[49] Campo-Tena L., Larmour S.R., Pereda N., Eisner M.P. (2024). Longitudinal Associations Between Adolescent Dating Violence Victimization and Adverse Outcomes: A Systematic Review. Trauma Violence Abus..

[50] Exner-Cortens D., Eckenrode J., Rothman E. (2013). Longitudinal Associations Between Teen Dating Violence Victimization and Adverse Health Outcomes. Pediatrics.

